# Vermicompost as an alternative substrate to peat moss for strawberry (*Fragaria ananassa*) in soilles culture

**DOI:** 10.1186/s12870-024-04807-0

**Published:** 2024-02-28

**Authors:** Mahsa Azizi Yeganeh, Ali Asghar Shahabi, Ali Ebadi, Vahid Abdossi

**Affiliations:** 1 Department of Horticultural Science and Agronomy, Science and Research Branch, Islamic Azad University, Tehran, Iran; 2 Soil and Water Research Department, Agricultural Research, Education and Extension Organization (AREEO), Isfahan Agricultural and Natural Resources Research and Education Center, Isfahan, Iran; 3 Department of Horticulture and Landscape Architecture, College of Agriculture and Natural Resources, The University of Tehran, Karaj, Iran

**Keywords:** Camarosa, Cultivar, Hydroponic, Selva, Vitamin C

## Abstract

**Background:**

Consecutive droughts and quantitative and qualitative reduction of surface and underground water resources have caused an increase in greenhouse and hydroponic cultivation for most garden crops, including strawberries, in Iran. On the other hand, most of the inputs of greenhouse crops in Iran are imported. To possibility of replacing vermicompost with peat moss under hydroponic cultivation, an experiment was done in a split plot based on randomized complete blocks design in three replications in Isfahan (Iran) Agricultural and Natural Resources Research Center in 2019. The main treatment was substrate at four levels included different levels of vermicompost (30 and 50%) and peat moss (30 and 50%) in combination with perlite and sub-treatment were Selva and Camarosa cultivars.

**Results:**

The results showed that Camarosa cultivar and Selva cultivar in (perlite/ peat moss 50:50) and Selva cultivar in (perlite / vermicompost 70:30) had maximum yield.

Leaf number and chlorophyll index were maximum in Camarosa cultivar in peat moss substrates. Strawberry cultivars had the highest root fresh weight, the content of vitamin C and total soluble solids (TSS) in substrates containing vermicompost. Camarosa cultivar in (perlite / peat moss50:50) and Selva cultivar in (perlite /vermicompost 50:50) had maximum root dry weight. Also, the highest number of inflorescences was related to substrates containing peat moss and (perlite /vermicompost 70:30). Maximum amount of fresh and dry weight of shoots were observed in (perlite/ peat moss70:30). Selva cultivar had more inflorescences (16.5%) than Camarosa cultivar and Camarosa cultivar produced more fresh and dry weight of shoots (16.5%, 23.01%) than Selva cultivar.

**Conclusion:**

Expriment results highlighted the importance of considering both main and sub-treatments in agricultural research, as they interacted to influence various growth and yield parameters. 50% vermicompost treatment combined with perlite had a positive impact on plant growth and in quality index such as vitamin C content and TSS was highest. while the choice of cultivar affected different aspects of plant development. Selva cultivar was known to be more tolerant to salinity caused by vermicompost. Vermicompost is local and more economical, also salt resistant cultivars are recommended in a controlled (30%) amount of vermicompost.

## Background

The cultivation of is an important agricultural practice worldwide. Farmers and researchers constantly seek innovative techniques to enhance crop productivity and quality. One such approach is the use of organic amendments like vermicompost and peat moss, which have demonstrated positive effects on plant growth and yield. Additionally, the selection of appropriate strawberry cultivars is crucial for optimizing outcomes. Strawberry (*Fragaria* × *ananassa* Duch.) is one of the most popular soft berry fruits [45]. Now, they are as a matter of functional food with numerous health benefits such as source of natural antioxidants, such as carotenoids, phenolics, vitamins, anthocyanins, and flavonoids [15, 31].

Food production by soilless culture method has become more attractive than traditional soil culture due to its advantages, such improved quality control over the growth environment and reduce of uncertainties related to soil, water, and nutrient availability [31].

Peat moss itself is a type of organic material derived from partially decomposed plant matter found in peat bogs. It is widely used in horticulture and gardening due to its excellent water-holding capacity, aeration properties, low pH, low bulk density, low nutrient content, and useful cation exchange capacity and ability to improve soil structure [11].

While peat moss has been widely used, there is growing concern about the sustainability of its extraction from peat bogs. As an alternative, compost-based substrates, and other organic materials are being explored as substitutes for peat moss in horticulture [32]. Vermicompost is one of domestically produced organic substrates that is important in agriculture sector, especially for production of greenhouse products. In recent years, use of vermicompost in horticulture as a high-use growing substrate has increased [15].

Organic composts exhibit qualities similar to peat in terms of porosity, aeration, and water holding capacity. They are renewable resources that are produced locally [21, 33].

Vermicompost is used as a suitable alternative to chemical fertilizers in various crops. Vermicomposting is a non-thermophilic process that converts organic waste materials into valuable fertilizer through the combination of worms and mesophilic microbes [33]. Vermicompost contain substances that control plant growth, such as humic acids, auxins, gibberellins, and cytokinins which control a variety of processes related to plant growth and yield [10].

There are some reports in the previous study on utilization, vermicompost in cultivation. The highest yield (408.04 g plant-1), fruit weight (18.5 g), Tss (8.33%), pH (3.95%) and total sugar ((Glucose + Fructose) mg 100 g − 1) in strawberry (*Fragaria vesca* L.) plants were obtained with the use of vermicompost (250 kg da − 1) compared to the control group and the use of chemical fertilizers [34].

Khatiwada [14] reported that shoot, root, and leaf growth characteristics were significantly affected by the application of vermicompost in strawberry (*Monterey cultivar*) plant.

In an experiment conducted by [39], the flower initiation was recorded at vermicompost (50%) also the highest fruit yield and highest soluble solid content at the time of harvest was recorded with treatment vermicompost (100%) and (50%).

In other experiment application of 100% vermicompost with other substrate was found most effective in vegetative attributes as well as yield per hectare of Strawberry fruit [41].

In an experiment conducted on strawberries, vermicompost in in a relatively low dose (equivalent to 170 kg N/ha) had a positive effect on the yield. Also, the quality of strawberry fruit such as total soluble solids, total anthocyanins, antioxidant activity of the fruit, and a lower concentration of total acid improved significantly [10].

The purpose of this study is to replace vermicompost instead of peat moss in Iran. Due to economic sanctions, using vermicompost in Iran can indeed be more economical than peat moss for several reasons: Local production, Lower cost, Sustainable waste management and Soil health benefits. In addition to being more budget-friendly, vermicompost also offers several benefits, such as being a sustainable and organic source of nutrients, improving soil structure and promoting beneficial microbial activity. These advantages contribute to enhanced plant growth and yield, making vermicompost a favorable choice for many farmers and growers.

## Results

### Yield

**Fig. 1:**
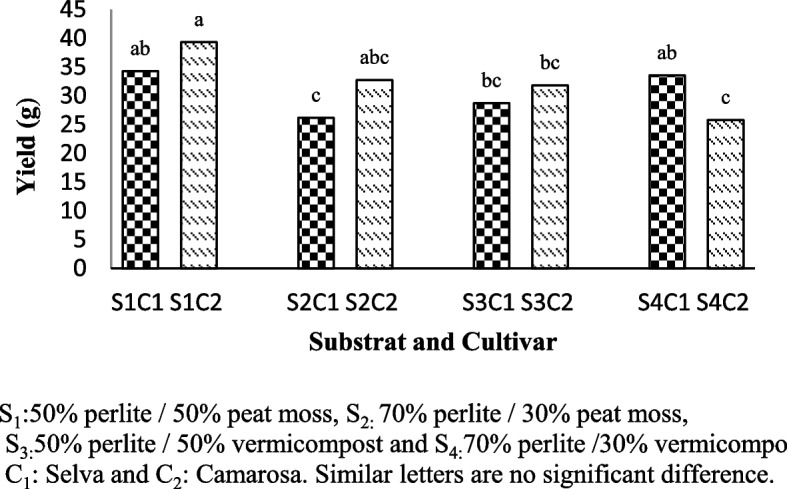
Yield of Selva and Camarosa in Vermicompost and Peat moss combined with Perlite

Yield of the two cultivars of strawberry was significantly affected by the substrate and interaction between substrate and cultivars (Table 1). Higher values of yield were recorded on Camarosa cultivar in the substrate with 50% perlite/ 50% peat moss (S1C2:39.31 gr) and on Selva cultivar with no significant difference in 50% perlite / 50% peat moss and 70% perlite /30% vermicompost (S1C1:34.26gr and S4C1:33.53gr) (Fig. 1).

**Table 1:** Analysis of variance substrate and cultivar on some physiological and biochemical traits of Selva and And Camarosa cultivar

Source of variance	Df	Mean sum of squares									
		Yield	Leaf	Inflorescence	Shoot fresh weight	Shoot dry weight	Root fresh weight	Root dry weight	Chlorophyll index	Total Soluble Solid	Vitamin C
SUBSTRATR (S)	3	74.16**	1.39**	0.13*	114.92**	12.79**	3.23^ns^	0.48^ns^	14.34**	0.57**	104.99^**^
Error	6	7.97	0.19	0.02	6.51	0.66	13.19	1.31	1.39	0.06	80.8
Cultivar (C)	1	18.28^ns^	1.17^ns^	0.34**	92.53*	17.96*	8.21^ns^	0.008^ns^	14.95**	0.47**	277.44**
S × C	3	62.89^*^	3.01^**^	0.08^ns^	23.47^ns^	1.94^ns^	7.95^*^	1.92^*^	3.59	0.18*	55.28^*^
Error	8	14.3	0.49	0.03	13.31	2.61	1.73	0.33	0.86	0.04	14.62
Coefficient of variation (%)		11.99	8.09	10.97	14.50	19.16	17.46	15.01	1.97	3.87	6.07

ns * and ** indicates non-significant, significant at *P* ≤ 0.05 and *P* ≤ 0.01, respectively

### The number of leaves

**Fig. 2:**
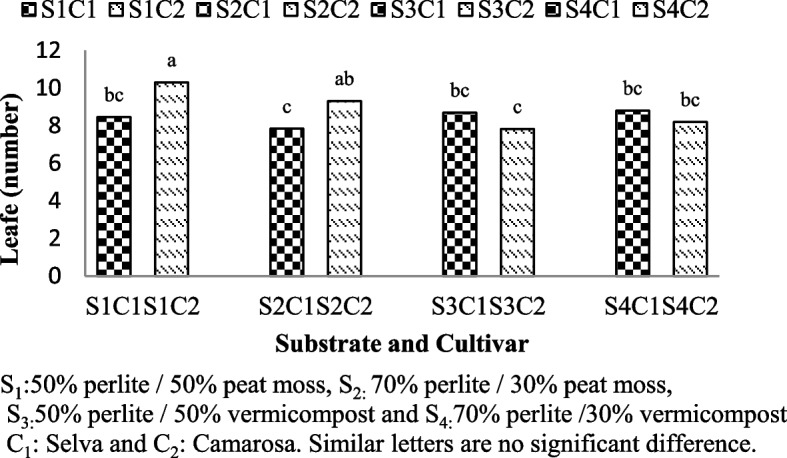
Leaf number of Selva and Camarosa in Vermicompost and Peat moss combined with Perlite

Leave numbers of strawberry were significantly affected by the substrate and interaction between substrate and cultivars (Table 1). Maximum number of leaves were recorded in Camarosa cultivar grown with 50% perlite/50% peat moss and 70% perlite/30% peat moss (S1C2: 10.3 and S2C2:9.31) (Fig. 2).

### Inflorescences

Inflorescences of strawberry significantly influenced by substrates and cultivars (Table 1). The highest number of inflorescences was related to substrates containing peat moss (S1 and S2) and 70% perlite / 30% vermicompost (S4) (Table 2). Selva cultivar produced more inflorescences (16.5%) than Camarosa (Table 2).

**Table 2:** Inflorescence, shoot fresh weight and shoot dry weight of Selva and Camarosa as affected by substrate and cultivar

Substrate	Inflorescence	Shoot fresh weight(g)	Shoot dry weight(g)
50% perlite / 50% peat moss (S1)	1.75^a^	8.85^b^	2.92^b^
70% perlite / 30% peat moss (S2)	1.61^ab^	10.12^a^	3.40^a^
50% perlite/ 50% vermicompost (S3)	1.40^b^	6.69^c^	2.22^c^
70% perlite / 30% vermicompost (S4)	1.51^ab^	7.86^bc^	2.72^b^
Cultivar			
Selva (C1)	1.69^a^	7.73^b^	2.52^b^
Camarosa (C2)	1.45^b^	9.04^a^	3.10^a^

Similar letters indicate no significant difference

### Fresh and dry shoot of weight

Fresh and dry shoot of weight significantly influenced by substrates and cultivars (Table 1). Maximum amount of fresh and dry weight of shoots were observed in 70% perlite/30% peat moss (S2) (Table 2). Camarosa cultivar produced more fresh and dry weight of shoots (16.5%, 23.01%) than Selva cultivar (Table 2).

### Fresh and dry root weight

**Fig. 3:**
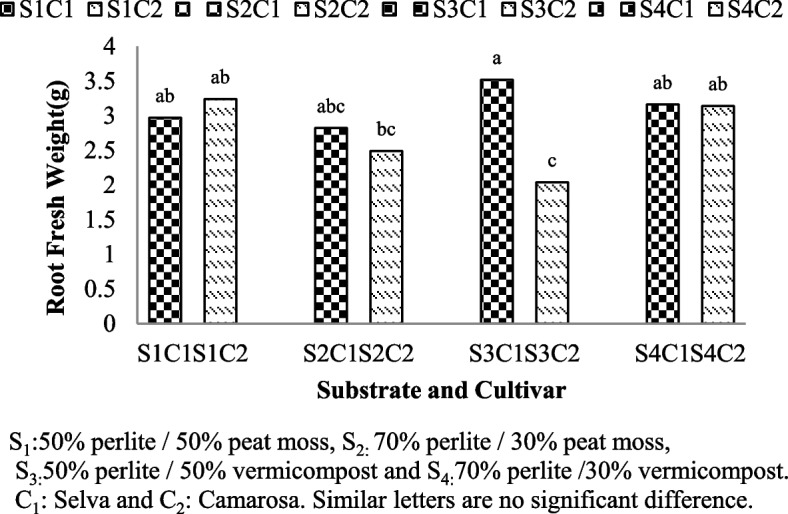
Root fresh weight of Selva and Camarosa in Vermicompost and Peat moss combined with Perlite

**Fig. 4:**
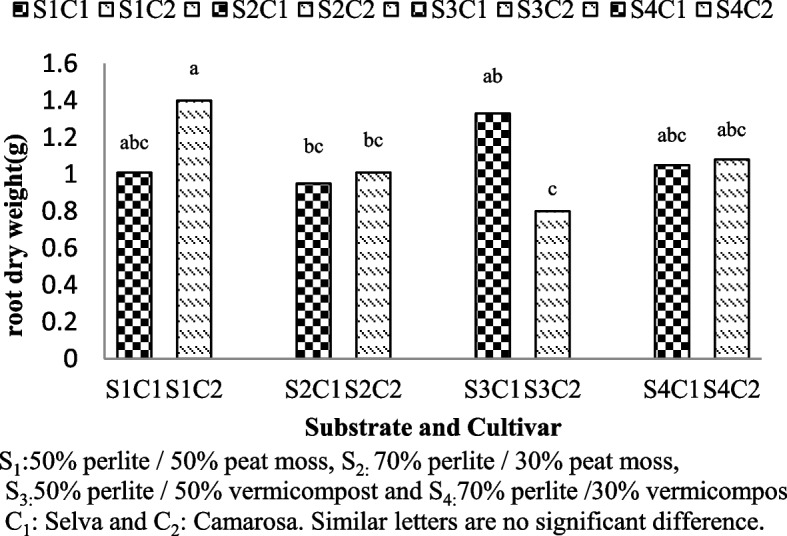
Root dry weight of Selva and Camarosa in Vermicompost and Peat moss combined with Perlite

Fresh and dry root of weight were significantly affected by interaction between substrate and cultivars (Table 1). Regarding to the measurement of root weight, higher fresh weight of roots was measured in Selva grown with 50% perlite/50% vermicompost (S3C1:3.52 gr) (Fig. 3), dry weight of roots in Camarosa cultivar grown with 50% perlite/50% peat moss and dry weight of roots in Selva cultivar grown with 50% perlite/50% vermicompost (S1C2:1.4 and S3C1:1.33) with no significant differences were recorded (Fig. 4).

### Chlorophyll

**Fig. 5:**
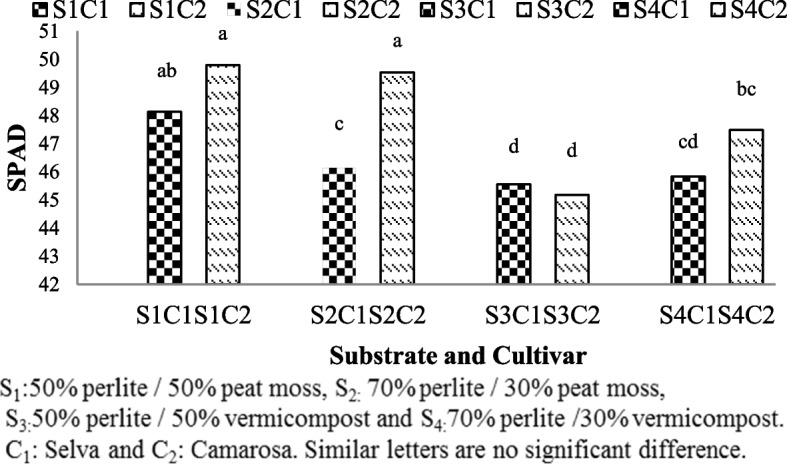
Chlorophyll index of Selva and Camarosa in Vermicompost and Peat moss combined with Perlite

Chlorophyll content significantly affected by substrate and cultivar and interaction between substrate and cultivars (Table 1). Levels of chlorophylls observed in Camarosa cultivar was higher in substrates containing peat moss (S1C2: 49.79 and S2C2:49.54) (Fig. 5). Both cultivars of strawberries grown in substrates with vermicompost did not have chlorophyll index as much as cultivars grown in substrate with peat moss (Fig. 5).

### Total Soluble Solid (TSS)

**Fig. 6:**
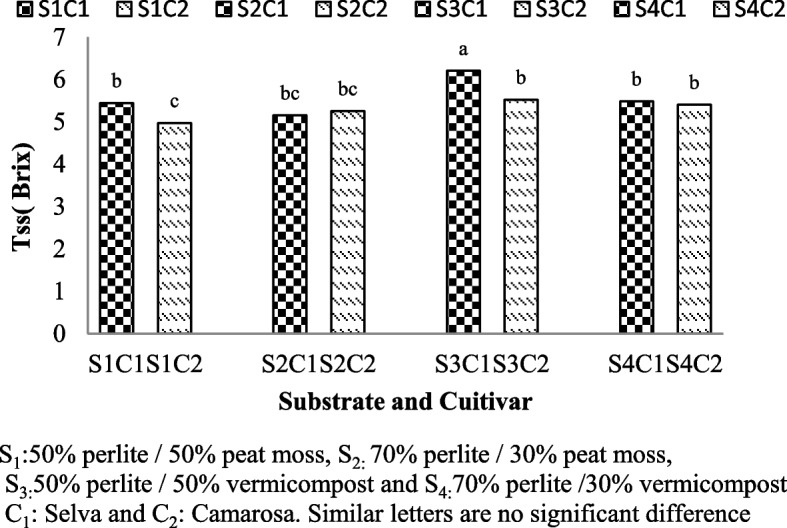
Total soluble solid of Selva and Camarosa in Vermicompost and Peat moss combined with Perlite

Total soluble solid showed significant differences between cultivars grown in substrate with peat moss and vermicompost (Table 1). Selva cultivar had the highest amount of total soluble solids in 50% perlite/50% vermicompost (S3C1: 6.2 Brix) (Fig. 6).

### Vitamin C contents

**Fig. 7:**
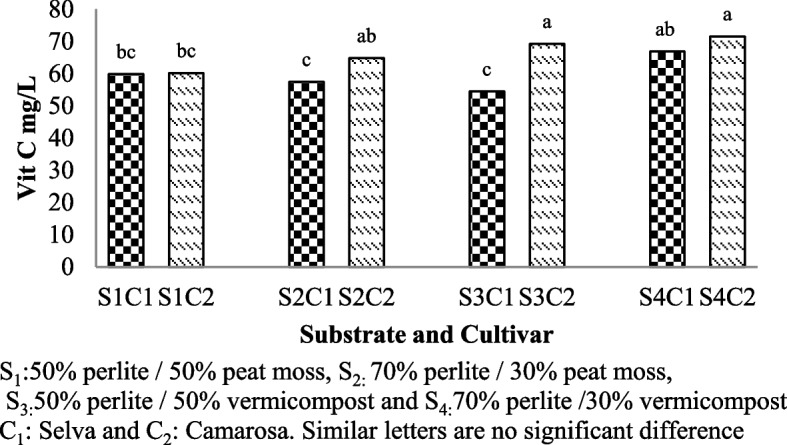
Vitamin C contents of Selva and Camarosa in Vermicompost and Peat moss combined with Perlite

Vitamin C contents of strawberries were significantly affected by the substrate, cultivars and interaction between them used (Table 1). An increase of vitamin C content was measured in Camarosa cultivar grown in the substrate based in vermicompost (S3C2:69.21mg/l and S4C2:71.52 mg/l) (Fig. 7).

## Discussion

### Yield

According to the result Selva cultivar had the highest yield in (perlite / peat moss 50:50) and (perlite / vermicompost 70:30) and the results in these two substrates were not significantly different from each other. This result showed that the substrate containing 30% vermicompost can be as effective as peat moss in increasing yield. vermicompost in low doses has a similar yield to peat moss because low doses of vermicompost contain lower levels of salts. High salt content in soil can negatively affect plant growth and yield. Increased yield by vermicompost in the results of research related to Marigold(*Tagetes*) [30], Eggplant (*Solanum melongena*)[25], Cucumber ( *Cucumis sativus*)[9], Okra( *Abelmoschus esculentus*) [6], Potato (*Solanum tuberosum*) [17] and Strawberry (*Fragaria ananassa*) [40] Is also visible. The positive role of vermicompost in this case can be explained by the fact that the presence of N, P, K in vermicompost, as well as micro and essential elements in it, was effective in increasing fruit weight and subsequent increase in yield, [23] are coordinated. Also, vermicompost reduces the accumulation of nutrients and increases the uptake by the plant by the coordination and balance that it creates between the release of nutrients and absorption, and consequently the plant yield increases [47].

### The number of leaves

Whereas the substrates with vermicompost had not the highest number of leaves, leaf production, in addition to depending on the type of cultivar, is affected by factors such as light, nutrients and growth factors. Peat moss can improve the cohesion of the root system and substrate [20], also the presence of growth hormones such as auxin in it [27] and creating a fine texture of the soil that increases water holding capacity [37], it can cause vegetative growth including the growth of leaves. In this trait, vermicompost could not produce as many leaves as peat moss. This may be because peat moss often improves retention, which is very effective in vegetative growth while vermicompost increases the fertility of the growing environment [3].

### Inflorescences

Although the plants grown in the peat moss substrates had more inflorescences, but the substrate with 30% vermicompost (S4) was able to produce the same number of flowers as the substrate with 30% peat moss (S2), without any significant difference between the two substrates (Table 2). Vermicompost, which is the product of composting organic materials using earthworms, has been shown to have positive effects on plant growth, including the improvement of inflorescences (flowers and flower clusters) in various plant species.The results of other researchers can be seen in positive effect of vermicompost in increasing the number of flowers in Marigold [30], Lilies (*Lilium*) [18] and Strawberry [7]. Increasing the level of nitrogen [10] and microbial activity following the addition of vermicompost leads to root expansion and greater uptake of nutrients, water and photosynthesis and ultimately leads to better flowers and shoots [12]. In addition, the presence of substances that affect plant growth, such as plant growth hormones and humic acids, has been suggested as a possible factor that helps increase plant growth [29]. The higher inflorescence production of Selva cultivar in vermicompost substrate could be attributed to several factors. Selva cultivar may have genetic characteristics that make it more responsive to the nutrients and conditions provided by vermicompost, resulting in increased inflorescence production compared to Camarosa cultivar or they might be better adapted to the specific growing conditions where they are cultivated. Additionally, the cultivation practices used for Selva cultivars might be optimized to encourage more inflorescence development compared to Camarosa cultivars. The type of cultivar also affected the number of inflorescences per plant, which [5] obtained a similar result.

### Fresh and dry shoot of weight

Fresh and dry shoot weight depends on plant species [3]. The plants grown in the vermicompost substrate did not have the highest fresh and dry weight of shoot. But fresh and dry weight of shoot in 50% perlite / 50% peat moss (S1) and 70% perlite / 30% vermicompost (S4) were without significant differences from each other (Table 2). Improving the growth of the branches may be related to improving the water condition of the branches. Loss of transpiration of leaves and water uptake by roots directly affects the water status of the branches. Peat moss has high porosity and higher water holding capacity than vermicompost which provides permanent and accessible moisture for plant and stimulates the growth of shoots [14]. In addition, peat contains different types of auxin (Indole acetic acid) hormones that cause vegetative growth in plants [27]. In 70% perlite /30% vermicompost (S4), when increasing perlite and increasing porosity and ventilation, shoot growth increased somewhat (Table 2). Stem weight with application of vermicompost can be seen in Lilium [22] and Pak Choi [28].

### Fresh and dry root weight

The increase in fresh and dry weight of roots can be attributed to several factors that may have positively influenced root growth and development. Root growth is a complex process influenced by various environmental, genetic, and physiological factors. The fresh and dry weight of the roots depends on the plant species [3]. The Selva cultivar might possess specific genetic traits that promote robust root growth. This would allow the plant to take up essential nutrients in vermicompost more effectively from the growing medium, leading to healthier and heavier roots. Vermicompost can play a significant role in increasing the fresh and dry weight of roots in plants. Increasing the absorption of essential macro and micro nutrients in vermicompost leads to improved root growth. When vermicompost is applied, it releases these nutrients gradually, providing a steady and balanced supply of nutrients to the plants. This nutrient availability promotes healthy root growth and encourages the development of a robust root system. In addition, the large amount of humic acid and the presence of auxin in vermicompost leads to the proliferation and elongation of secondary roots and increase the total length of the root surface [15, 29]. Vermicompost also improves the condition of the substrate and its water holding capacity by affecting the physical, chemical and biological properties of the substrate [16], which is effective in root expansion. The improved substrate structure allows roots to penetrate the substrate more easily and encourages lateral root development. Significant activity of microorganisms in vermicompost that convert ammonium nitrogen to nitrate can also increase root diameter and increase fresh and dry weight of roots [35]. Also, these microbes contribute to the development of a healthy rhizosphere (root zone) by promoting nutrient cycling and improving nutrient uptake by roots. Vermicompost has been previously shown to result in increase weight of root in pea (*Pisum sativum*) [13], dry weight of root in pepper (*Capsicum*) and strawberry [7], tomato (*Solanum lycopersicum*) and cucumber [8] and fresh weight of root in *Lilium longiflorum* [22].

### Chlorophyll

The chlorophyll index in plants refers to the relative amount of chlorophyll present in the leaves, which is an indicator of photosynthetic activity and plant health. While both peat moss and vermicompost can be used as substrate to enhance plant growth, there are specific differences in their effects on the chlorophyll index.

when comparing the chlorophyll index in peat moss and vermicompost directly, vermicompost is more likely to result in a higher chlorophyll index due to its nutrient content, particularly nitrogen [29], but in this experiment, vermicompost could not have maximum chlorophyll index. Peat moss / perlite ratio (1: 1) is used as a standard substrate in most culture systems [2]. Therefore, the high chlorophyll content in strawberries grown in peat moss can be attributed to properties such as low pH, good texture and good water retention ability that is effective in providing high nutrient status and creating suitable conditions for growth [37].

The pH of the growing medium can influence nutrient availability to plants. Peat moss is known to have a slightly acidic pH [37], which can enhance the availability of certain nutrients like iron, which is essential for chlorophyll synthesis. Also, peat moss might have been provided a more suitable nutrient balance for chlorophyll synthesis in this experiment.

### Total Soluble Solid (TSS)

Total soluble solid and total acidity are the main sensory and taste factors in strawberry fruits. Fruit brix values depend on variety, cultivation system and harvesting stage [44]. Different plant cultivars have varying genetic traits that can affect their ability to accumulate and transport sugars and other soluble solids within their tissues. The Selva cultivar may have genetic characteristics that promote higher sugar accumulation, leading to increased total soluble solids.

The increase in TSS and total sugar in strawberries is associated with the rapid conversion of starch and pectin metabolites to soluble compounds and the rapid translocation of sugars from the leaves (source) to the growing fruit (sink) [16]. Vermicompost is a nutrient-rich organic fertilizer, and its application can influence the availability of nutrients to plants which can help to increase and synthesize sugar in fruits [23]. Vermicompost contains beneficial microorganisms that can enhance nutrient uptake and nutrient use efficiency in plants [35]. These microorganisms may have positively influenced the Selva cultivar's ability to absorb and utilize nutrients, leading to higher total soluble solids. Also, the potassium present in vermicompost increases the accumulation of sugar in berries and the balance is N, P, K, which is essential for the proper accumulation of sugar in fruits [34]. In strawberry [16, 19, 40], cucumber [9], tomato [1] and cabbage (*Brassica oleracea*) [26] the highest soluble solids were in vermicompost.

### Vitamin C content

Vitamin C, which has a water-soluble structure, is a very important vitamin for protecting human health due to the presence of antioxidants in it. Vitamin C content depends on cultural practices, light intensity, weather conditions [34]. Different strawberry cultivars can have varying genetic traits that influence their nutrient composition, including vitamin C content. The Camarosa cultivar might naturally have a higher capacity for vitamin C synthesis and accumulation. Ascorbic acid is a biologically active form of vitamin C, the content of which can change with respect to growth and storage conditions and genetics of cultivars [46] Vitamin C content is also affected by plant nutrition, water availability and light intensity [48]. Vermicompost provides a wide range of nutrients for storage and microbial activity, and provides most nutrients available, such as N, P, Ca and exchangeable potassium for plant [40]. Having micronutrients such as iron, zinc, copper and manganese as well as high water and food storage capacity [42], can be considered as one of the reasons for the high level of vitamin C in the substrate based in vermicompost. The results of the present experiment with the results of other researchers on increase of vitamin C by vermicompost in tomatoes [1, 4], potatoes [38], cabbage [26], is consistent.

## Conclusion

Based on the results obtained, the following conclusions can be drawn: The choice of substrate composition significantly influenced the growth and development of the strawberry plants.

### Yield potential

The highest yield was observed in the Camarosa cultivar when grown in peat moss substrate. For Selva cultivar, the combination of 70% perlite and 30% vermicompost resulted in higher yields. These findings suggest that substrate composition plays a crucial role in determining the yield potential of different cultivars.

### Cultivar-Specific responses

The two cultivars, Selva and Camarosa, exhibited variations in their responses to different substrate compositions. For instance, the Camarosa cultivar showed higher leaf number and chlorophyll index in peat moss-based substrates, while the Selva cultivar demonstrated higher fresh and dry root weights in the combination of perlite and vermicompost.

### Quality attributes

The substrate composition also affected various quality attributes of the strawberries. The highest vitamin C content was observed in the Camarosa cultivar when grown in vermicompost, while the Selva cultivar exhibited higher total soluble solids (TSS) levels in the combination of perlite and vermicompost. These results highlight the potential of vermicompost-based substrates in enhancing fruit quality attributes. In conclusion, the choice of substrate composition in hydroponic strawberry cultivation has a significant impact on plant growth, yield, and quality attributes. Vermicompost can serve as a viable alternative to peat moss, with different cultivars exhibiting varying responses to substrate compositions.

## Methods

The present study was done during 2019–2020 in Isfahan Agricultural and Natural Resources Research Center greenhouse (Isfahan Province, Iran) by hydroponic culture. Strawberry seedlings (Camarosa and Selva) were received from the agricultural center Kurdistan. For preparation of volumetric mixtures in order to eliminate the possible salinity of vermicompost culture substrate, leaching was performed on this substrate. No operation was performed on perlite and peat moss culture substrate. The experiment was performed in split plots as a randomized complete block design with 3 replications. The main treatment of culture substrate at four levels included volumetric mixture of 50% perlite and 50% peat moss (S_1_), 70% perlite and 30% peat moss (S_2_), 50% perlite and 50% vermicompost (S_3_), and 70% perlite and 30% vermicompost (S_4_) and sub-treatment were Camarosa and Selva.

The analysis of vermicompost and peat moss substrates are given in Table 3 and 4.

**Table 3:** properties of vermicompost used in the experiment

Vermicompost	K_2_O %	P_2_O_5_%	N %	OM %	PH	EC (ds/m)
	1.30	1.70	1.05	49.0	7.3	1.2

**Table 4:** Properties of Peat moss used in the experiment

Peat moss	EC	pH Value H_2_O	Amount of added fertilizer NPK fertilizer 14:10:18	Recommended use
	45 ms/m (± 25%)	5.5- 6.5	1.5 kg/m^3^	70 L

Peat moss: Produced by Klasmann -Deilmann GmbH40744Geeste.Germany

The field capacity of used substrates was like this: FC: 50% perlite / 50% peat moss (S1):203%, FC: 70% perlite /30% peat moss (S2):145%, FC: 50% perlite/ 50% vermicompost (S3):59.5%, FC: 70% perlite / 30% vermicompost (S4):42.5%

For hydroponic planting, pots with an opening diameter of 18 cm were used and the nourishment method was open. Nutrient solution (including macro and micro nutrients from Table 5) needed by the plants was prepared in barrels (100 L) and 200 cc per pot was provided to the plants daily. The roots were immersed in Captan + Mancozeb fungicide solution for 2 s and immediately, 3 seedlings were planted in each pot. The greenhouse temperature was adjusted at 18 °C (night) and 25 °C (day) and relative humidity (60%) was considered constant for the treatments.

**Table 5:** Nutrients of soilless culture of strawberry in a general substrate [24]

Growth stage	Mg/l												Salinity ds/m
	N	P	K	Mg	Ca	S	Fe	Mn	Zn	B	Cu	Mo	
Vegetative Growth	207	65	184	58	221	77	6.5	2.6	0.25	0.7	0.07	0.05	2
Fruit Growing	182	82	301	58	148	77	6.5	2.6	0.25	0.7	0.07	0.05	2

**Fig. 8:**
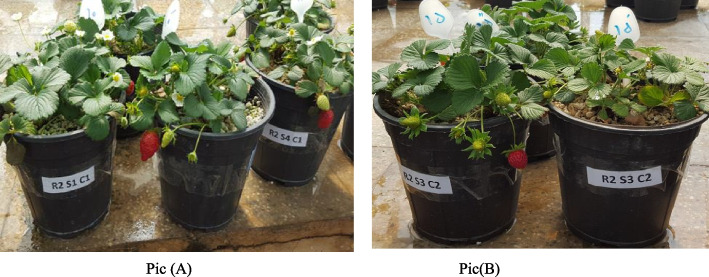
pots of strawberry cultivation Selva (Pic **A**) and Camarosa (Pic **B**) in 4 volumetric mixtures. Abbreviations used: S_1_:50% perlite / 50% peat moss, S_2:_ 70% perlite / 30% peat moss, S_3:_50% perlite / 50% vermicompost and S_4:_70% perlite /30% vermicompost. C_1_: Selva and C_2_: Camerosa

During the growing season, some of the most important morphological and biochemical indicators properties of cultivated pots (Fig. 8 Selva (Pic A) and Camarosa (Pic B) were estimated. Data acquisition started when the plants gave new leaves and continued until the end of the experiment. Traits were measured every 7 to 10 days and at the end an average was reported for each trait.

### Total weight of fruits (yield)

When the fruits of the plants were fully ripe and had a uniform red color, the fruits were harvested and weighed with a digital scale model Electronic Compact Scale SF_400C.

### Fresh/ dry weight of shoots and roots:

The roots and aerial parts were dried separately for 72 h in an oven at a temperature of 85 °C and then weighed with a scale with an accuracy of 0.01 [43].

### Number of leaves and of inflorescences

Number of leaves and of inflorescences were counted.

### Chlorophyll index

3 to 4 leaves were randomly selected from each plant and then measured using a chlorophyll meter model SPAD-502, Minolta, Osaka, Japan. The average value of leaf chlorophyll index was read in three replicates for each treatment.

### Total soluble solids

Total soluble solids was displayed by refractometer device model PR-101Atago Co. Ltd., Japan, respectively.

### Vitamin C content

Vitamin C content was determined by titration of potassium iodide [36].

### Statistical analysis

The data were analyzed using MSTAT-C software and means were compared by Duncan's multiple range test at the probability level of 5%. Excel software was also used to draw the graphs.

## Data Availability

The data that support the findings of this study are available from the corresponding author upon reasonable request.
