# Correction: Vermicompost as an alternative substrate to peat moss for Strawberry (*Fragaria ananassa*) in soilles culture

**DOI:** 10.1186/s12870-024-04909-9

**Published:** 2024-04-01

**Authors:** Mahsa Azizi Yeganeh, Ali Asghar Shahabi, Ali Ebadi, Vahid Abdossi

**Affiliations:** 1 Department of Horticultural Science and Agronomy, Science and Research Branch, Islamic Azad University, Tehran, Iran; 2 Soil and Water Research Department, Agricultural Research, Education and Extension Organization (AREEO), Isfahan Agricultural and Natural Resources Research and Education Center, Isfahan, Iran; 3 Department of Horticulture and Landscape Architecture, College of Agriculture and Natural Resources, The University of Tehran, Karaj, Iran

**Correction: BMC Plant Biol 24, 149 (2024)**

10.1186/s12870-024-04807-0

Following publication of the original article [1], author spotted an error in one of the author’s family name in the author list and in the body under Statement about the plants section. The correct family names are highlighted in **bold typeface**.

Incorrect author name:

Vahid Abdoosi

Mehsa Azizi Yeganeh

Correct author name:

Vahid **Abdossi**

**Mahsa** Azizi Yeganeh

The corrections do not affect the overall result or conclusion of the article. The original article has been corrected.
